# Supplementary material for: Fast and robust analog in-memory deep neural network training
Source: Nat Commun. 2024 Aug 20;15:7133. doi: 10.1038/s41467-024-51221-z (PMC11335942; doi:10.1038/s41467-024-51221-z)
Supplement: Supplementary file 1 — Supplementary Information [file 41467_2024_51221_MOESM1_ESM.pdf]

Fast and robust analog in-memory deep neural network training

Malte J. Rasch[‡1, 2], Fabio Carta[1], Omobayode I. Fagbohungbe[1], and Tayfun Gokmen[§1]

[1]IBM Research, TJ Watson Research Center, Yorktown Heights, NY USA
[2]Sony AI, Zürich, Switzerland

July 13, 2024

Contents

A Supplementary Figures **2**

B Algorithms **7**

C Supplementary Methods **10**
C.1 deep neural network (DNN) training simulation details 10

List of Figures

1 Example physical implementation of in-memory MVM 2
2 In-memory training algorithm AGAD 2
3 Example device traces 3
4 Algorithm implementation details 3
5 Example DNN training traces 4
6 Vision transformer training traces 4
7 Effect of longer transfer periods (n_s) 5
8 Retention requirement of $\breve{A}$ 5
9 Time for gradient computation and weight update depending on available digital compute 6

[‡]Correspondence: malte.rasch@gmail.com
[§]Correspondence: tgokmen@us.ibm.com

A Supplementary Figures

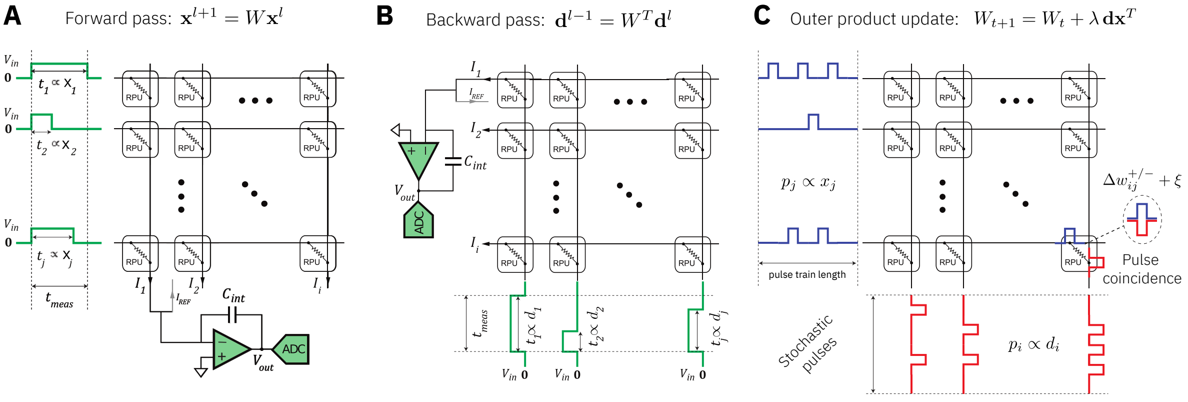

Supplementary Figure 1: **Example physical implementation of in-memory matrix-vector multiplication (MVM)** for forward pass (plot **A**), backward passes (plot **B**) and direct (and naïve) outer product weight update using stochastic pulsing according to [1] (plot **C**).

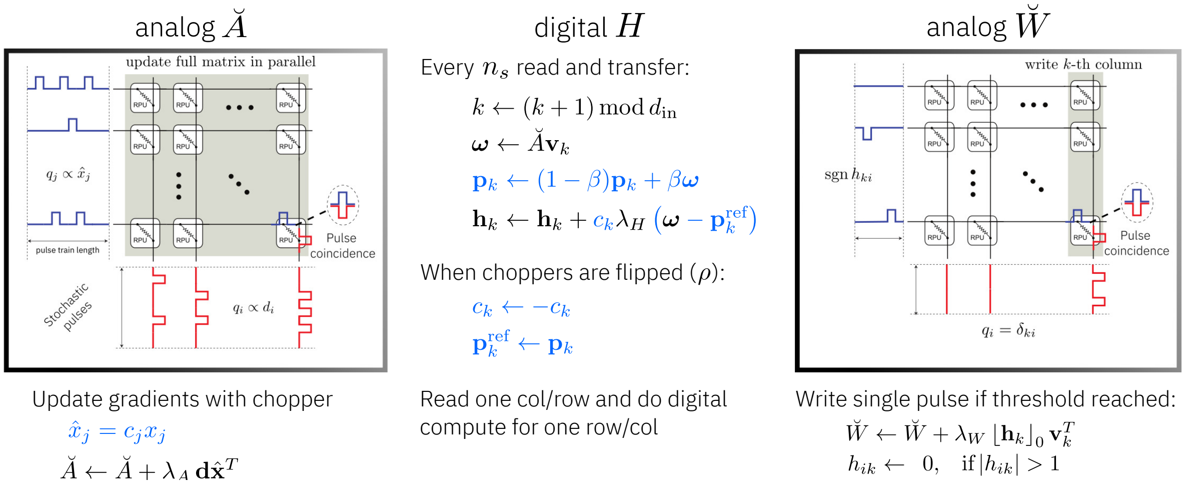

Supplementary Figure 2: **In-memory training algorithm AGAD.** As described in Alg. 3, the algorithm is based on the overall principle of TTv2 (see Fig. 4.6.1 for an illustration) but adds additional digital compute. The blue color equation indicate the additional compute in comparison to TTv2. Note that the reference matrix $\breve{R}$ (see Fig. 4.6.1) is not used here as $\breve{A}$ is read directly without a differential read.

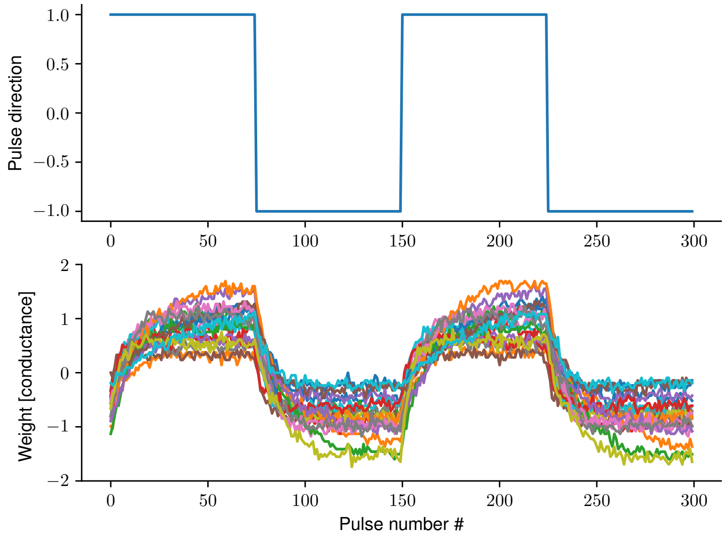

Supplementary Figure 3: **Example device traces**. Response traces of the simulated material used ($n_{\text{states}} = 20$). Upper plot shows the pulsing pattern. Lower plot shows the response of 20 devices (colors). Note that significant asymmetry, device-to-device variation and cycle-to-cycle variation is present.

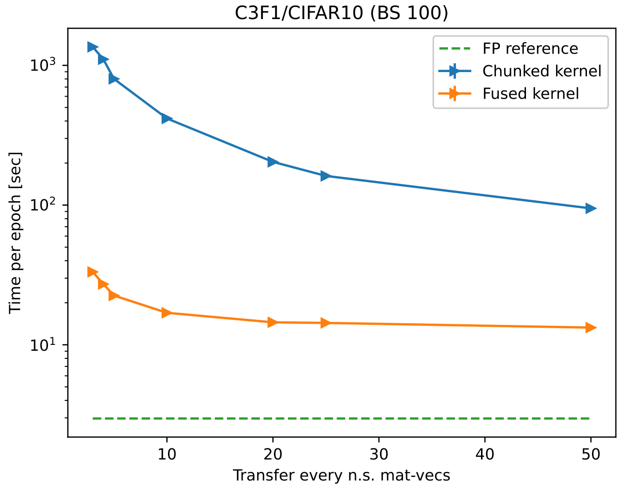

Supplementary Figure 4: **Algorithm implementation details**. Runtime of our improved CUDA kernel design for TTv2 (fused kernel) in comparison to the existing chunked kernel of the open source toolkit AIHWKit. The TTv2 algorithm was already implemented in the toolkit, however, we here improved on its GPU implementation by an runtime speed-up of up to 40× by using a fused CUDA-kernel design (instead of the existing chunked kernel. We also implemented a custom-designed device model, which explicitly subtracts the symmetry point. The fused kernel is also used for our algorithms c-TTv2 and AGAD. Here the time for one epoch on a standard small CNN on the CIFAR-10 dataset [2] is shown. The runtime is evaluated for different n_s parameters (compare to Supplementary Fig. 2). Note that our fused design improves the speed dramatically, from > 1000 seconds to only 30 seconds in the best case.

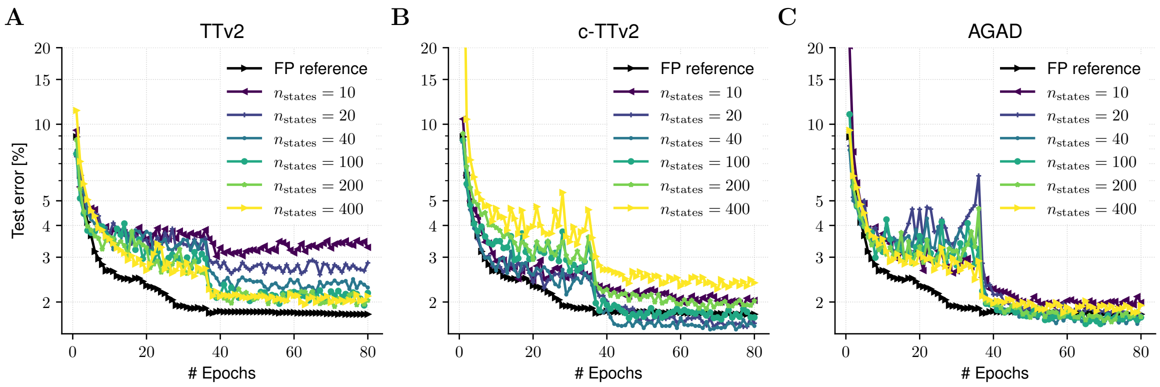

Supplementary Figure 5: **Example DNN training traces**. Comparison of the convergence of the learning algorithms. Here the test error is calculated after each training epoch and shown versus training epochs. Learning rate is reduced in step-wise manner which causes a rapid decrease in test error around 40 epochs (10-fold reduction in learning rate). Note that overall convergence is somewhat slower in comparison to the FP reference, however, the required training epochs are on the same order of magnitude. Same experiments as in Fig. 5 A (3-FC / MNIST), however, here the reference offset variation is set to $\sigma_r = 0.0$ to focus on the rate of convergence only. Only traces for the best hyper-parameter settings for varying number of device states are shown. **A**: TTv2 algorithm. **B**: c-TTv2 algorithm. **C**: AGAD algorithm. See Supplementary Methods Sec. C.1.1 for details on the experiments.

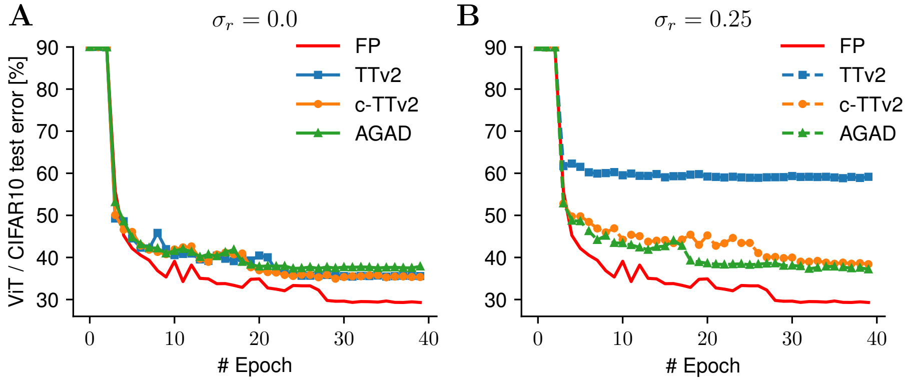

Supplementary Figure 6: **Vision transformer training traces**. Comparison of the convergence of the learning algorithms for a vision transformer on CIFAR 10. Here the test error is calculated after each training epoch and shown versus training epochs. **A**: No reference offset variation is present, $\sigma_r = 0.0$. The FP SGD test error is shown as a comparison. Note that no image augmentation is used here. **B**: Repeat with reference offset variation set to $\sigma_r = 0.25$. Note that the TTv2 algorithm has significantly more test error, whereas c-TTv2 and AGAD algorithms are less or not affected, respectively. See Supplementary Methods Sec. C.1.4 for details on the experiments.

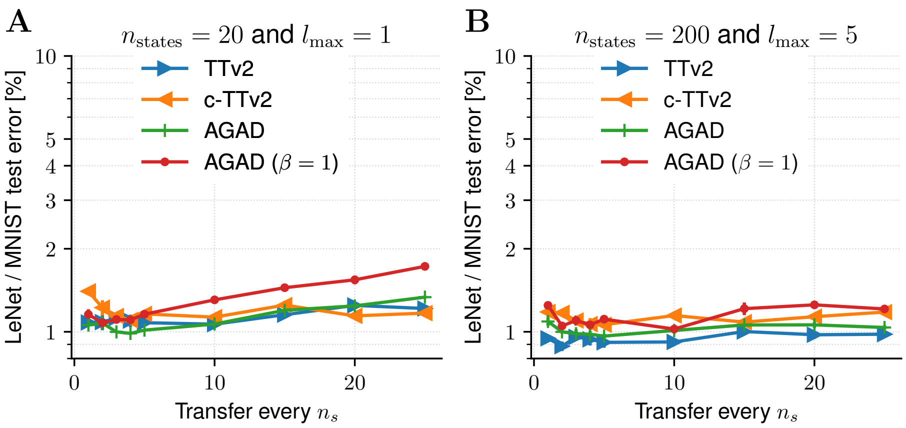

Supplementary Figure 7: **Effect of longer transfer periods (n_s).** LeNet DNN is used on the MNIST data set for illustration. Plot **A** sets number of states to $n_{\text{states}} = 20$ and the maximal number of pulses per input onto $\breve{A}$ to $l_{\max} = 1$, whereas in plot **B** sets $n_{\text{states}} = 200$ and $l_{\max} = 5$. **A, B**: Test errors are plotted for various settings of n_s and shows that similar accuracy can be reached. Note that throughput is increased for larger n_s (as additional digital operations are less often executed). There is slight drop in accuracy for larger n_s and smaller n_{states} as the coarser devices can hold accumulated information for less long. This is seen for AGAD if the leaky average estimation of P is omitted (red dotted line, $\beta = 1$). Parameters: as in Fig. 3, except $\gamma_0 = 10000$ and $\lambda_A = 0.1$.

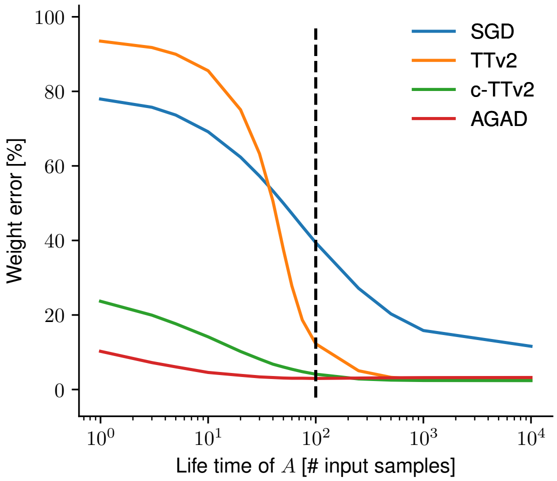

Supplementary Figure 8: **Retention requirement of $\breve{A}$.** Same weight programming experiment as in Fig. 4, however, with $\delta = 0.05$. The resulting weight error is plotted for different setting of the life time l of $\breve{A}$. The lifetime is defined as decay with rate $1 - 1/l$ towards a random position in the conductance range for each device. The decay is applied after each input sample, so that l is measured in unit of input samples. Note that weight error recovers if the life time is on the order or larger than the period of weight transfer $n_s n = 100$ (dashed line). Parameters: otherwise as in Fig. 4.

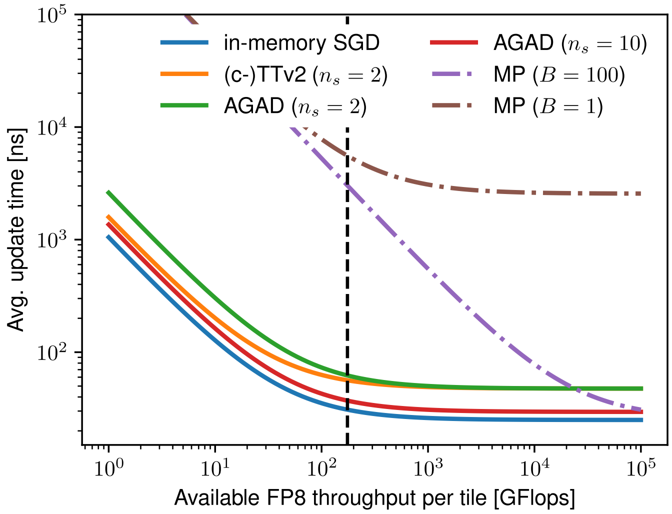

Supplementary Figure 9: **Average time for gradient computation and weight update depending on available digital compute**. Lines show different algorithms and parameter settings, such as mini-batch size B and transfer period n_s as indicated in the legend. When limited digital compute is available, the in-memory weight update algorithms (solid lines) vastly outperform the digital gradient accumulation approach (dashed-dotted lines). Note that the speed of the analog operations become limiting when enough digital compute is available. Other parameters: $l_{\text{avg}} = 5$, matrix size $N \times N$ with matrix size assumed to be $N = 512$. See Tab. 1 for the equations used to determine the expected runtime.

B Algorithms

Algorithm 1 Parallel in-memory update using dynamically adjusted stochastic pulsing as illustrated in Supplementary Fig. 1 C.

input data vector x_i, size n, gradient vector d_j, size m, weight matrix $\breve{w}_{ij}$, average pulse size at SP δ, learning rate η, max pulses $l_{\max}$, pulse vectors q_i and p_j
output Updated analog weight matrix $\breve{w}_{ij}$
  set $m_x \leftarrow \max_i |x_i|$ and $m_d \leftarrow \max_j |d_j|$
  set $\kappa \leftarrow \frac{\eta m_x m_d}{\delta}$
  set $l \leftarrow \min(l_{\max}, \lceil\kappa\rceil)$ and $\gamma \leftarrow \sqrt{\frac{\kappa}{l}}$
  set $a \leftarrow \max(\gamma, \frac{\kappa}{l_{\max}})$ and $b \leftarrow \min(\gamma, 1)$
  for $k = 1$ **to** l **do**
    set all $q_i \leftarrow 0$ and all $p_j \leftarrow 0$
    for $i = 1$ **to** m **do**
      Draw random number $\xi \in \mathcal{U}(0, 1)$
      if $\xi < \frac{a|d_i|}{m_d}$ **then**
        set $q_i \leftarrow \text{sgn}(d_i)$
      end if
    end for
    for $j = 1$ **to** n **do**
      Draw random number $\xi \in \mathcal{U}(0, 1)$
      if $\xi < \frac{b|x_j|}{m_x}$ **then**
        set $p_j \leftarrow \text{sgn}(x_j)$
      end if
    end for
    {Send pulse vectors p_j and q_i to update $\breve{w}_{ij}$}
    for $i = 1$ **to** m **do**
      for $j = 1$ **to** n **do**
        if $p_j \neq 0$ and $q_i \neq 0$ **then**
          set $\breve{w}_{ij} \leftarrow \breve{w}_{ij} + \Delta\breve{w}_{\text{sgn}(q_i p_j)}\,(\breve{w}_{ij}\,|\,\boldsymbol{\theta})$
        end if
      end for
    end for
  end for

Algorithm 2 Chopped-TTv2 (c-TTv2) algorithm. The original TTv2 algorithm [3] can be recovered by setting $\rho = 0$ (ie. without choppers). However, we here use in both algorithms the dynamic learning rate scaling as described in Sec. 4.6.

---

input analog matrices $\breve{A}$, $\breve{R}$, $\breve{W}$ (with pulse updates according to Eq. 4), digital matrix H, learning rates λ_A and λ_H, transfer period n_s, chopper probability ρ
output converged analog weights $\breve{w}_{ij}$
  set choppers $c_j \leftarrow 1$
  set counters s and k to 0 and digital matrix $h_{ij} \leftarrow 0$
  call Program reference $\breve{R}$ to the SP of $\breve{A}$: $\breve{r}_{ij} \leftarrow \breve{a}^*_{ij}$
  repeat
    call start SGD iteration for mini-batch {(using $\breve{w}_{ij}$ as weights) until the update of $\breve{w}_{ij}$ is required}
    for all inputs $\mathbf{x}$ and $\mathbf{d}$ to be updated onto $\breve{W}$ **do**
      set λ_A according to Eq. 15
      set λ_H according to Eq. 14
      call Alg. 1 to update $(c_j x_j)$ and $\mathbf{d}$ onto $\breve{A}$ with learning rate λ_A
      set $s \leftarrow s + 1$
      if $s = n_s$ **then**
        set $k \leftarrow k + 1 \mod n$
        set $s \leftarrow 0$
        set $q_i \leftarrow 0$ for all i
        call the analog MVM: $\mathbf{y} \leftarrow (\breve{A} - \breve{R})\,\mathbf{v}_k$
        for $i = 1$ **to** m **do**
          set $h_{ik} \leftarrow h_{ik} + c_k \lambda_H y_i$, and $q_i \leftarrow 0$
          if $|h_{ik}| > 1$ **then**
            set $q_i \leftarrow \text{sgn}(h_{ik})$
            set $h_{ik} \leftarrow 0$
          end if
        end for
        {Use pulse vector q_i to update row k of $\breve{W}$}
        for $i = 1$ **to** m **do**
          if $q_i \neq 0$ **then**
            set $\breve{w}_{ik} \leftarrow \breve{w}_{ik} + \Delta\breve{w}_{\text{sgn}(q_i)}\,(\breve{w}_{ik}\,|\,\boldsymbol{\theta})$
          end if
        end for
        {Flip the choppers}
        draw random number $\xi \in \mathcal{U}(0, 1)$
        if $\rho < \xi$ **then**
          set $c_k \leftarrow -c_k$
        end if
      end if
    end for
    call finish SGD iteration
  until convergence is reached

Algorithm 3 Analog Gradient Accumulation with Dynamic reference (AGAD) algorithm. Using dynamic on-the-fly reference point computation. Note that here the analog matrix $\breve{R}$ is not needed. compare also to Supplementary Fig. 2 for an illustration.

input analog matrices $\breve{A}$, $\breve{R}$, $\breve{W}$ (with pulse updates according to Eq. 4), digital matrices H, P, and P^{ref}, learning rates λ_A, λ_H transfer period n_s, chopper frequency ρ, leaky-average time-scale β
output converged analog weights $\breve{w}_{ij}$
  set counters s, k and t to 0 and all choppers $c_j \leftarrow 1$
  set digital matrices h_{ij}, p_{ij} and p_{ij}^{ref} to all 0
  repeat
    call start SGD iteration
    for all inputs $\mathbf{x}$ and $\mathbf{d}$ to be updated onto $\breve{W}$ **do**
      set λ_A according to Eq. 15
      set λ_H according to Eq. 14
      call Alg. 1 to update $c_j x_j$ and d_i onto $\breve{a}_{ij}$ using learning rate λ_A
      set $s \leftarrow s + 1$
      if $s = n_s$ **then**
        set $s \leftarrow 0$
        set $t \leftarrow t + 1$
        set $q_i \leftarrow 0$ for all i
        set $k \leftarrow k + 1 \mod n$
        call analog MVM: $\mathbf{y} \leftarrow \breve{A}\, \mathbf{v}_k$
        for $i = 1$ **to** m **do**
          set $p_{ik} \leftarrow (1 - \beta)\, p_{ik} + \beta y_i$
          set $h_{ik} \leftarrow h_{ik} + c_k\, \lambda_H \left(y_i - p_{ik}^{\text{ref}}\right)$
          if $|h_{ik}| > 1$ **then**
            set $q_i \leftarrow \text{sgn}(h_{ik})$
            set $h_{ik} \leftarrow 0$
          end if
        end for
        {Use pulse vector q_i to update row k of $\breve{W}$}
        for $i = 1$ **to** m **do**
          if $q_i \neq 0$ **then**
            $\breve{w}_{ik} \leftarrow \breve{w}_{ik} + \Delta\breve{w}_{\text{sgn}(q_i)}\, (\breve{w}_{ik}\,|\,\boldsymbol{\theta})$
          end if
        end for
        {Flip chopper and set p_{ij}^{ref} as follows}
        if $t \mod \left\lceil \frac{1}{\rho} \right\rceil = 0$ **then**
          set $c_k \leftarrow -c_k$
          for $i = 1$ **to** m **do**
            set $p_{ik}^{\text{ref}} \leftarrow p_{ik}$
          end for
        end if
      end if
    end for
    call finish SGD iteration
  until convergence is reached.

C Supplementary Methods

C.1 DNN training simulation details

Here we describe the details of the DNN training simulations done in Fig. 5.

C.1.1 3-FC / MNIST

We use a 3-layer fully connected DNN on the MNIST data set with sigmoid activations and hidden sizes of 255 and 127 as described in [1]. For the analog MVM (forward and backward pass), we use essentially the standard settings of AIHWKit, which includes output noise (0.5 % of the quantization bin width), quantization and clipping (output range set to 20, output noise to 0.1, and input and output quantization to 8 bit). It uses the noise and bound management techniques as described in [4]. To adjust the output range to the DNN range, we add a learnable (scalar) FP factor after each crossbar output, which is updated with standard momentum SGD (0.9 momentum term).

We train for 80 epochs and schedule the learning rate into 3 steps (35, 35, and 10 epochs), with reduction factor of 0.1. Starting learning rate is set to 0.05 (0.1 for FP) and batch size is 10. Additionally, we set $n_s = 1$ and $\gamma_0 = 10000$ and confirmed by a test run that it was a reasonable setting. We then run a grid of simulations and report the average test accuracy over the last 3 training epochs. The grid was the same for each condition, namely we simulated the settings of $\lambda_A = 0.05, 0.1, 0.2, 0.5, 1.0, 2.0$. For each condition the best test error (averaged over the last 3 epochs) is reported in the figure. The number of states was varied as indicated in the figure (see Fig. 5; parameter δ). Other parameters are set as reported in Fig. 3. The chopper probability was set to $\rho = 0.1$, where random switching was used for c-TTv2 and regular switching for AGAD as described in the algorithms (see Alg. 2 and Alg. 3, respectively).

For Fig. 5 A, we additionally varied the learning rate for each condition, and took the better one of the two $\lambda = 0.05$ or $\lambda = 0.025$. This resulted in 432 training simulations for this subplot alone. In Supplementary Fig. 5, we show test error traces with training epoch for the hyper-parameter combinations that had the lowest test error (averaged over the last 20 epochs, where the learning rate was already very small), and show a trace for each learning algorithm and the number of states is varied additionally (compare to Fig. 5 A). Here we show the case with no reference offset variation $\sigma_r = 0.0$. Note that the learning dynamics is slightly slower but comparable to the floating point reference.

C.1.2 LeNet / MNIST

We follow [4] and use a variant of the LeNet5 [5] model architecture, which contains 2-convolutional layers, 2-max-pooling layers, and 2-fully connected layers, trained on the MNIST dataset [6]. The model training is performed using the same setting of AIHWKit for the analog MVM (forward and backward pass) and the same device model as described above (Supplementary Methods Sec. C.1.1).

The model is trained for 60 epochs using a batch size of 8 and $\gamma_0 = 10000$. Different learning rate λ values of 0.01, 0.02, 0.03, 0.04, and 0.05 are explored for $\sigma_r = 0.0$, and the learning rate with the best test accuracy value is picked for each method under consideration, which resulted in the values 0.03, 0.04, and 0.04 for TTv2, c-TTv2, and AGAD, respectively. The learning rate is scheduled into two steps (45 and 15 epochs) with a reduction factor of 0.10. The chopper probability (for the c-TTv2 and AGAD cases) is set to $\rho = 0.1$, with random switching used for c-TTv2 and regular switching for AGAD.

The initial values of λ_A and n_s are set to 1, and 1, respectively. Firstly, the value of λ_A is tuned until the best result is obtained. After that, the value of n_s is also tuned. This tuning sequence is encouraged as λ_A has more influence on the model performance as measured using the test accuracy than n_s. The combination of these hyper-parameter values that gives the best result is selected for all the methods. We find that $n_s = 1$ works best for all methods and set λ_A to 0.05, 0.1 and 0.075 for TTv2, c-TTv2, and AGAD, respectively. The test accuracy reported is the average over the test errors obtained after the last three training epochs.

The above obtained values of the λ_A and n_s hyper-parameters are then used to obtain the test accuracy for $\sigma_r = 0.05, 0.1, 0.25, 0.5, 1.0$ respectively, and the resulting test accuracy is used to generate the plot in Fig. 5 C.

C.1.3 LSTM / War & Peace

We use an Long short-term memory (LSTM) network composed of 2 stacked LSTM blocks with hidden vector size of 64 followed by a fully connected layer, as described in [7]. We trained this network on the War and Peace (WP) novel. The dataset is split into a training set and test set with 2,933,246 and 325,000 characters, respectively, and a total vocabulary of 87 characters.

We trained the network using the AIHWKit for 100 epochs and schedule the learning rate into 3 steps (50, 40, and 20 epochs) with a reduction factor of 0.1. The starting learning rate is set to 0.1, batch size to 16, sequence length to 100, $\gamma_0 = 10000$ and chopper probability (for the c-TTv2 and AGAD cases) is set to $\rho = 0.1$, with random switching used for c-TTv2 and regular switching for AGAD.

We run multiple simulations starting from the condition with reference offset variation $\sigma_r = 0$. We explore different learning rate $\lambda = 0.05, 0.1, 0.75$, different settings for $n_s = 1, 3$ and for $\lambda_A = 0.05, 0.1, 0.5, 1.0$ and take the best combination of parameter for each algorithm. With the selected parameters, we then perform the simulations for various reference offset variations and take the loss of the last epoch to generate the plot in Fig. 5 D.

C.1.4 Vision transformer / CIFAR10

The Vision Transfomer (ViT) model used here [1] is based on the shifted patch tokenization (SPT) and locality self-attention (LSA) methods proposed in [8], which are generic and effective add-on modules that are easily applicable to various ViTs. ViT models operate on a sequence of image embeddings obtained by dividing its input images into patches, which are then treated as a group of learnable embeddings. These modules were introduced to solve the lack of locality inductive bias, which is critical if the model can learn from small datasets. The model architecture can be broadly divided into three blocks. The first block is responsible for converting the images into patches and contains one linear layer, and the SPT module is implemented here. This block is also responsible for adding the token embedding vector and the positional embedding to the patches. The second block is the transformer block, which mainly contains the multi-attention head and the feedforward neural network responsible for the model performance. The LSA module is implemented in each of the transformer's attention blocks. This ViT model contains four transformer blocks and, hence, 4 attention and LSA modules. The last block is the MLP head, which can be considered the classifier block containing one linear layer. This model contains about 4,337,642 trainable parameters and 18 linear layers in total. Parameters of all linear and convolution layers are assumed in analog in-memory computing (AIMC), other layers, such as the normalization layers are computed in FP.

We trained the network using the AIHWKit for 40 epochs without image augmentation and reduce the learning rate with reduction factor of 0.1 if a plateau is reached. Because of much longer simulation time for this larger model, we only used standard parameter settings without exploring hyper-parameters as in the other smaller DNNs. We set $n_s = 1$ and $\lambda_A = 0.075$ which worked reasonably in earlier experiments. The number of device states is set to $n_{\text{states}} = 200$, the batch size is 8, $\gamma_0 = 10000$ and chopper probability (for the c-TTv2 and AGAD cases) is set to $\rho = 0.01$.

Results Consistently with the results on the benchmark DNNs, we found that when no noise is added to the symmetry point reference, the classification error is 36.1% (0.6% standard deviation (SD) over 5 trials), 35.9% (0.4% SD), and 37.5% (0.6% SD) for TTv2, c-TTv2, and AGAD, respectively, however, if a SD of $\sigma_r = 0.25$ is added (compare to Fig. 5 B-D), then test errors become 59% (SD 0.1%), 37.9% (SD 0.4%), and 37.3% (SD 0.3%), respectively (see also Supplementary Fig. 6). This again shows the

[1] The model used in this work follows the recipe used in the repository `https://github.com/kentaroy47/vision-transformers-cifar10`

susceptibility of TTv2 for an inaccurate symmetry point reference value, in contrast to the proposed algorithmic improvements c-TTv2 and AGAD.

Here, the FP test error is 29.3% using our setup (without image augmentation, 40 epochs, no transfer learning). Note again that due to simulation time limitations for this relatively large DNN, no hyper-parameter optimization was performed for the analog training algorithms for this experiment, which might explain the remaining gap to the FP accuracy. Also, image augmentation with longer training runs, as well as transfer learning, is likely to improve the obtainable accuracy considerably, which, however, is beyond the focus of the current study.

References

[1] T. Gokmen and Y. Vlasov, "Acceleration of deep neural network training with resistive cross-point devices: Design considerations," *Frontiers in neuroscience*, vol. 10, p. 333, 2016.

[2] A. Krizhevsky, G. Hinton, *et al.*, "Learning multiple layers of features from tiny images," 2009.

[3] T. Gokmen, "Enabling training of neural networks on noisy hardware," *Frontiers in Artificial Intelligence*, vol. 4, pp. 1–14, 2021.

[4] T. Gokmen, M. Onen, and W. Haensch, "Training deep convolutional neural networks with resistive cross-point devices," *Frontiers in neuroscience*, vol. 11, p. 538, 2017.

[5] Y. LeCun, L. Bottou, Y. Bengio, and P. Haffner, "Gradient-based learning applied to document recognition," *Proceedings of the IEEE*, vol. 86, no. 11, pp. 2278–2324, 1998.

[6] L. Deng, "The mnist database of handwritten digit images for machine learning research," *IEEE Signal Processing Magazine*, vol. 29, no. 6, pp. 141–142, 2012.

[7] T. Gokmen, M. J. Rasch, and W. Haensch, "Training LSTM networks with resistive cross-point devices," *Frontiers in neuroscience*, vol. 12, p. 745, 2018.

[8] S. H. Lee, S. Lee, and B. C. Song, "Vision transformer for small-size datasets," *arXiv preprint arXiv:2112.13492*, 2021.
